# ERK2 Is a Promoter of Cancer Cell Growth and Migration in Colon Adenocarcinoma

**DOI:** 10.3390/antiox13010119

**Published:** 2024-01-17

**Authors:** Alessia Parascandolo, Giulio Benincasa, Francesco Corcione, Mikko O. Laukkanen

**Affiliations:** 1 Department of Translational Medical Sciences, University of Naples Federico II, Via Pansini 5, 80131 Naples, Italy; al.parascandolo@libero.it; 2 Italo Foundation, 20146 Milano, Italy; giulio.benincasa@pinetagrande.it; 3 Clinica Mediterranea, 80122 Naples, Italy; francesco.corcione@clinicamediterranea.it; 4 Center for Experimental Endocrinology and Oncology (IEOS), CNR-IEOS, Via Pansini 5, 80131 Naples, Italy

**Keywords:** colon cancer, ERK1/2, SOD3, migration, invasion, proliferation

## Abstract

ERK1/2 phosphorylation is frequently downregulated in the early phase of colon tumorigenesis with subsequent activation of ERK5. In the current work, we studied the advantages of ERK1/2 downregulation for tumor growth by dissecting the individual functions of ERK1 and ERK2. The patient sample data demonstrated decreased ERK1/2 phosphorylation in the early phase of tumorigenesis followed by increased phosphorylation in late-stage colon adenocarcinomas with intratumoral invasion or metastasis. In vitro results indicated that SOD3-mediated coordination of small GTPase RAS regulatory genes inhibited RAS-ERK1/2 signaling. In vitro and in vivo studies suggested that ERK2 has a more prominent role in chemotactic invasion, collective migration, and cell proliferation than ERK1. Of note, simultaneous *ERK1* and *ERK2* expression inhibited collective cell migration and proliferation but tended to promote invasion, suggesting that ERK1 controls ERK2 function. According to the present data, phosphorylated ERK1/2 at the early phase of colon adenocarcinoma limits tumor mass expansion, whereas reactivation of the kinases at the later phase of colon carcinogenesis is associated with the initiation of metastasis. Additionally, our results suggest that ERK1 is a regulatory kinase that coordinates ERK2-promoted chemotactic invasion, collective migration, and cell proliferation. Our findings indicate that ROS, especially H_2_O_2_, are associated with the regulation of ERK1/2 phosphorylation in colon cancer by either increasing or decreasing kinase activity. These data suggest that ERK2 has a growth-promoting role and ERK1 has a regulatory role in colon tumorigenesis, which could lead to new avenues in the development of cancer therapy.

## 1. Introduction

The four main MAPKs responding to stress signaling, p38 MAPK, extracellular-signal-regulated kinase (ERK1/2), Big MAP kinase (ERK5), and c-jun N-terminal kinase (JNK), have a pivotal function in cancer progression [1]. The kinase p38 MAPK has been shown to have a profound effect on the early phase of inflammation-associated colon tumorigenesis by stimulating inflammatory cytokine and chemokine production, thereby modulating the innate immune system and contributing to the initiation of tumorigenesis [2]. Correspondingly, it has been shown that the inhibition of p38 MAPK activates epidermal growth factor receptor ErbB3 with subsequent activation of MEK1/2-ERK1/2 independently of rat sarcoma (RAS) or rapidly accelerated fibrosarcoma (RAF) [3].

ERK1/2 activation is regulated by a complex network of tyrosine kinase receptors, cellular kinases, small GPTases and their regulatory proteins, protein scaffolds, phosphatases, mutations in the oncogenes, and reactive oxygen species (ROS) [4,5]. Activation of ERK1/2 signal transduction is initiated by cell membrane receptors which recruit adapter molecules (e.g., growth factor receptor-bound protein 2 (Grp2)) and small GPTase regulatory proteins that stimulate GTP transfer to small GPTases, such as RAS. The GTP-bound RAS activates downstream kinase RAF, which further transmits the signal to mitogen-activated protein kinase kinase 1/2 (MEK1/2) and ERK1/2. MEK1/2 phosphorylates ERK1/2 at Thr202 and Tyr204 causing a conformational change in the kinase that enables the interaction of ERK1/2 with their downstream target molecules, e.g., ETS Like-1 protein (Elk-1) [6]. Phosphorylated ERK1/2 kinases stimulate growth, migration, survival, differentiation, and in certain cases also cell cycle arrest, depending on their cellular location and the downstream molecules they activate [2,7,8,9,10]. Dual phosphatases (DUSPs) regulate ERK1/2 activity through a negative feedback loop. DUSP expression, especially DUSP5 and DUSP6, is induced by ERK1/2 that subsequently results in inhibition of the kinase phosphorylation and thereby reduced DUSP expression [11].

ERK1/2 are intermediator molecules in signal transduction contributing to oncogene-induced senescence (OIS) [12], the transformation of primary cells [13], increased superoxide anion (O_2_^−^) and hydrogen peroxide (H_2_O_2_) levels by stimulating *nox1* mRNA synthesis, NOXO1 activation, and extracellular superoxide dismutase (SOD3) production [14]. SOD3 regulates O_2_^−^ levels at the cell membrane compartment concomitantly regulating its own expression levels; low substrate level inhibits the enzyme expression mRNA expression [15].

RAS-ERK1/2 feedback regulatory mechanisms contribute to redox balance through fine-tuning of small GTPase activity, consequently affecting the activation status of the signaling pathway. RAS-ERK1/2 signaling increases SOD3 expression thereby increasing H_2_O_2_ synthesis at the cell membrane. H_2_O_2_ migrates into the cells through aquaporin 3 channels thereby affecting the protein tyrosine phosphatase (PTP) activity and the expression of small GTPase regulatory gene guanine nucleotide-exchange factors (GEFs), GTPase-activating proteins (GAPs), and guanine nucleotide-disassociation inhibitors (GDIs) that regulate RAS activity. Increased ERK1/2 phosphorylation augments SOD3 expression, thereby forming a positive feedback loop [14,15,16,17].

In metastatic colon cancer, deregulation of the RAS-MEK1/2-ERK1/2 signaling pathway downstream of epidermal growth factor receptor by RAS-associated mutations confers a selective growth and survival advantage to tumor cells giving them an acquired resistance to anti-EGFR therapy [18].

Previous studies have demonstrated downregulation of ERK1/2 phosphorylation in colon tumorigenesis in adenomas and early phase adenocarcinomas [19,20,21], with a subsequent increase in ERK5 phosphorylation to maintain the progression of tumorigenesis [22] and the stem cell-like malignant phenotype of cancer cells [23].

In the current work, we studied the advantage of ERK1/2 inactivation for the progression of colon tumorigenesis. Our data corroborated previous observations [19] showing downregulation of the phosphorylation of ERK1/2 in adenomas and in early-phase adenocarcinomas which, based on our in vitro data, could be caused by ROS-mediated downregulation of the RAS-BRAF-MEK1/2-ERK1/2 signaling cascade. Simultaneous ERK1 and ERK2 expression significantly inhibited cell proliferation but it also enhanced in vitro cancer cell chemotactic invasion through extracellular matrix and metastasis observed in patients. In vitro and in vivo data suggested a more pronounced role for ERK2 tumorigenesis as compared to ERK1, which could function as a regulatory kinase promoting or inhibiting ERK2 function.

Consequently, our data may suggest that the downregulation of ERK1/2 phosphorylation is needed for initial colon tumor expansion, whereas the upregulation of ERK1/2 activation promotes metastasis.

## 2. Methods

### 2.1. Tissue Staining

Human adenocarcinoma sections were stained with ERK1/2 (Cell Signaling, Danvers, MA, USA) and hematoxylin–eosin (Sigma-Aldrich, St. Louis, MO, USA). The staining protocol was performed using Leica Bond Max Autostainer (Leica, Wetzlar, Germany). The clinical stage of the disease was determined using the TNM staging method (T = The size of the tumor (0–4), N = metastasis to lymph nodes, number of lymph nodes metastasized, M = metastasis to other organs). Tumor grading was conducted according to the WHO system, which is determined by the stage of undifferentiation of the cells, i.e., the number of abnormalities in the cellular phenotype. Colon cancer is usually divided into three grades: well-differentiated (low grade, G1), moderately differentiated (intermediate grade, G2), and poorly differentiated (high grade, G3) [24]. Patients older than 18 years who were surgically operated on for colon cancer were eligible to participate in the study. Ethical permissions for the study were approved by Clinica Mediterranea ethical committee, Naples, Italy, Monaldi Hospital ethical committee (Deliberazione del Direttore Generale n:o 1239), Naples, Italy, and by the University of Naples Federico II ethical committee (protocol number 394/19), Naples, Italy. Informed consent was obtained from patients participating in the study.

### 2.2. Mice

The mice study was a custom order study from Plasant Srl (Plasant Srl, Rome, Italy). BALB/c Nude Mice (Plaisant Srl, Rome, Italy) were xenografted with HCT116 cells transduced with *GFP* (n = 6 xenografts), *ERK1* (n = 6 xenografts), *ERK2* (n = 6 xenografts), or *ERK1/2* (n = 6 xenografts). To study the specific effect of individual ERK1 or ERK2 kinases, 1 × 10^6^ cells were used, which were the lowest number of cells resulting in tumorigenesis in GFP control animals. Tumor growth was measured by caliber twice a week for five weeks. Animal ethical permission was requested by Plaisant SRL and approved by the Italian Ministry of Health, Decreto ministeriale Nro. 15/20121-UT.

### 2.3. Cell Lines

Normal colon epithelial CCD841 cells (ATCC, Manassas, VA, USA) were cultured in EMEM/10% FBS/L-alanine-l-glutamine/penicillin–streptomycin (ATCC). DLD1 cells (ATCC) were cultured in RPMI/10% FBS/L-alanine-l-glutamine (Life Technologies, Grand Island, NY, USA), penicillin–streptomycin (Sigma, St. Louis, MO, USA). HCT116 cells (ATCC) were cultured in DMEM/10% FBS/L-alanine-l-glutamine/penicillin–streptomycin. Human colon cells were transduced with green fluorescence protein (*GFP)*, human *ERK1*, or *ERK2* lentiviruses (MOI 1) or RNAi viruses for the kinases (MOI 1) (ABM, Vancouver, Canada). Mouse embryonic fibroblasts (MEF) clones (MEF GFP, MEF SOD3 cl6, MEF SOD3 cl8, and MEF SOD3 cl5) [25] were cultured in αMEM (Mediatech, Manassas, VA, USA) supplemented with 10% FBS (Hyclone, Logan, UT, USA), non-essential amino acids (Mediatech, Manassas, VA, USA) and L-alanine-l-glutamine (Life Technologies, Grand Island, NY, USA), and penicillin–streptomycin (100 mg/L) (Sigma, St. Louis, MO, USA).

Normal colon CCD841 epithelial cells have a limited life span of less than 100 population doublings. DLD1 cells, derived from adenocarcinoma, are tumorigenic in nude mice, and carry activated oncogenes. The culture contains 0.2–0.4% of side population (SP) stem cells and 1% CD133 positive cells [26,27], indicating their undifferentiation status. HCT116 cells lack the differentiation capacity almost completely, form primary tumors in nude mice, and have been demonstrated to metastasize. Analysis of HCT116 has suggested that the cultures are made up of almost solely CD166-positive cancer stem cells, explaining their highly aggressive nature [27,28] (Supplemental Figure S1). MEF clones cl5, cl6, and cl8 were derived from individual mouse embryos transduced with an ecotropic retrovirus containing *GFP* marker gene or rabbit *sod3* cDNA [25,29].

### 2.4. Gene Expression Analysis

RNA isolated using an RNeasy mini kit (Qiagen, Hilden, Germany) was reverse transcribed to cDNA using a QuantiTect reverse transcription kit (Qiagen). Quantitative PCR was performed using SYBR green PCR master mix (Applied Biosystems, Foster City, CA, USA). Primers are listed in the Supplemental Table S1.

### 2.5. Western Blot Analysis

The proteins were isolated from the tissues and cells were homogenized in lysis buffer (50 mmol/L HEPES pH 7.5, 150 mmol/L NaCl, 10% glycerol, 1% Triton X-100, 1 mmol/L EGTA, 1.5 mmol/L MgCl_2_, 10 mmol/L NaF, 10 mmol/L sodium pyrophosphate, 1 mmol/L Na_3_VO_4_, 10 μg aprotinin/mL, and 10 μg leupeptin/mL) (Sigma). Antibodies used were: phospho-ERK1/2 (Thr202/Tyr204) (Cell Signaling), ERK1/2 (Cell Signaling, Danvers, MA, USA), phospho-MEK1/2 (Ser217/221) (Cell Signaling), MEK1/2 (Cell Signaling), phopho-ERK5 (Thr218/Tyr220) (Merck KGaA, Darmstadt, Germania), ERK5 (Cell Signaling), RAS (Cell Signaling), RAC (Cell Signaling), RHO (Cell Signaling), CDC42 (Cell Signaling), and TUBULIN (Cell Signaling). Intensity of Western blot bands was analyzed using ImageJ software version 1.53a.

### 2.6. Pull-Down Assay for MEF SOD3 Clones

Cells grown to 60% confluence were collected for pull-down analysis of small GTPase RAS, RAC, RHO, and CDC42. Cells were lysed using an ice-cold buffer containing 20 mM HEPES (pH 7.4), 0.1 M NaCl, 1% Triton X-100, 10 mM EGTA, 40 mM glycerophosphate, 20 mM MgCl_2_, 1 mM Na_3_VO_4_, 1 mM dithiothreitol, a mixture of protease inhibitors, and 1 mM phenylmethylsulfonyl fluoride. The lysates were gently shaken for 15 min with a purified GST-fusion protein containing the CRIB domain of PAK1 (p21 activated kinase) bound to glutathione-Sepharose beads. The mix was washed three times using lysis buffer. GTP-bound forms of RAS (Santa Cruz, Dallas, TX, USA) and RAC (Millipore, Burlington, MA, USA) were analyzed by Western blotting.

### 2.7. Growth Analysis

To analyze cell proliferation, 10,000 cells were seeded on 24-well plates in triplicates and counted daily for four days.

### 2.8. Matrigel Migration

To study the cellular invasion towards chemo-attractant using a transwell migration assay, 100 μL of Matrigel (Corning, Corning, NY, USA) at 1 mg/mL was added to the migration chambers (8 microns, BD, San Jose, CA, USA) and allowed to stabilize at room temperature for 30 min. To analyze the invasion, 50,000 cells were seeded on the Matrigel in a 5% FBS medium and left to migrate toward a 10% FBS medium for 48 to 72 h depending on the cell line characteristics. To detect the migrated cells, the Matrigel was removed from the chambers, cells were fixed with 7% paraformaldehyde (Sigma), washed with PBS, and stained with cristal violet (Sigma). Migrated cells were counted from the high-power microscope fields (Leica DMI3000B microscope and Leica Application Suite camera software, Leica Application Suite X 1.1.0.12420, Wetzlar, Germany). Naïve counterpart fibroblasts isolated from the same patients were used as TAFs as controls.

### 2.9. Wound Healing

For the collective cell migration assay, cells on 6-well plates (Corning) were grown to 60% confluency, their culture was broken with a scratch, and images (Leica) of the culture were taken at 24 h intervals. The distance between the edges of the scratch was measured for the calculation of the closure percentage.

### 2.10. Limitations of the Study

The main limitations of the study are as follows: (1) the similarities of ERK1 and ERK2 on cDNA (75.5% similarity in FASTA comparison of Genbank BC013992 and OM301623.1 sequences) and protein (84% similarity [30]) levels, and (2) different splice variants of the kinases. These factors may have an impact on the expression, regulation, and functions of the kinases. Each branch of the ERK1/2 signaling route contains one or more splice variant isoform with unique functions and cell type-specific expression [6].

### 2.11. Statistical Analysis

The experiments were repeated three times. *p*-values (* *p* < 0.05, ** *p* < 0.01, *** *p* < 0.001) were determined by two-tailed independent samples t-tests. Results are expressed as the mean ± SD.

## 3. Results

### 3.1. Inverse Correlation of ERK/2 Phosphorylation and Progression of Colon Tumorigenesis

**Figure 1:**
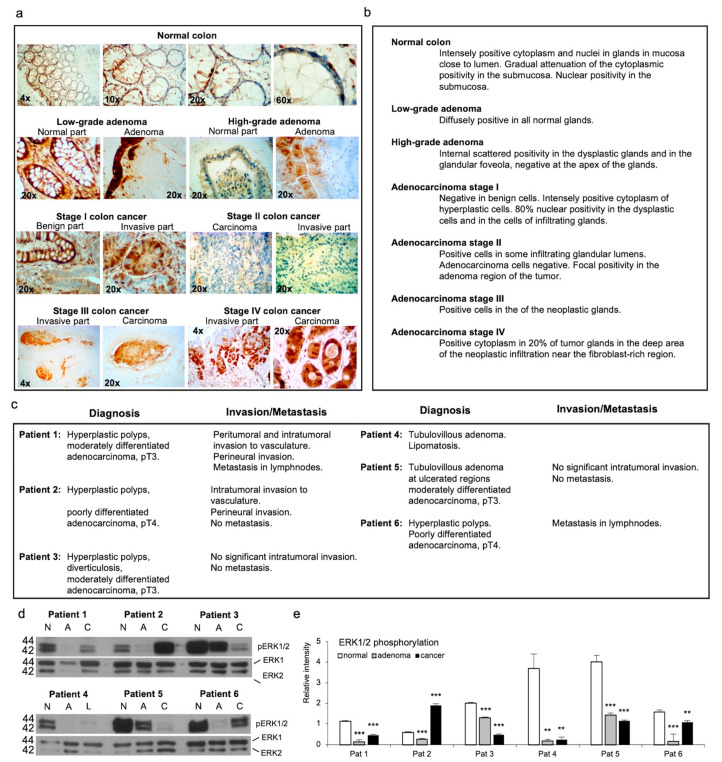
ERK1/2 staining in histological tissue sections. (**a**) ERK1/2 immunohistology staining of the normal colon, low- and high-grade adenoma, and stage 1–IV adenocarcinoma. Magnifications of the normal colon images are 4×, 10×, 40×, and 60×. Magnification of the low- and high-grade adenomas is 20×. Magnification of stage I and II colon cancer is 20×. Magnifications of stage III and IV colon cancer images are 4× and 20×. (**b**) Description of the staining data. Normal colon tissue close to the lumen of the colon was intensely positive suggesting a high endothelial cell proliferation rate. Positivity decreased in stages I–III. In stage III there was intensive staining in neoplastic glands. In stage IV the most intensive staining was observed in the infiltration areas near the fibroblast-rich region. (**c**) Diagnosis of patients used for Western blot analysis in panel d. Patient 1, patient 2, and patient 6 had intratumoral invasion or metastasis. Patient 3 had moderately differentiated adenocarcinoma without metastasis. Patient 4 had lipomatosis, and patient 5 had moderately differentiated adenocarcinoma without metastasis (**d**) Western blot analysis of patients. Normal tissue, adenoma, and adenocarcinoma tissues from each patient were used for the analysis. (**e**) Intensity analysis of phosphoERK1/2 staining from the Western blot. All patients demonstrated reduced ERK1/2 activation in adenomas. The reduction was further augmented in patients 3, 4, and 5. Patient 1, patient 2, and patient 6 showed increased phosphorylation when compared to adenomas. *p*-values are ** *p* < 0.01, and *** *p* < 0.001. To view this illustration in color, the reader is referred to the online version of this article.

A recent mouse model study emphasized the adaptability of colon cancer signaling suggesting increased ERK5 kinase phosphorylation to compensate for an abrogation of ERK1/2 activation in colon tumorigenesis in maintaining cell proliferation [22]. To dissect the characteristics of ERK1/2 kinase activation in colon tumorigenesis, human normal colon, adenoma, and adenocarcinoma tissue sections were stained with phosho-ERK1/2 antibody. Robust ERK1/2 phosphorylation in the normal mucosa, most prominently in the luminal regions, and markedly reduced phosphorylation of the kinase in adenomas and adenocarcinomas (Figure 1a,b) were observed.

Reduced activation of the kinases in adenomas and adenocarcinomas was confirmed by a Western blot analysis, thereby corroborating the previous observations (Figure 1d,e) [19,20,21]. We further demonstrated recovery of the ERK1/2 phosphorylation in patients diagnosed with advanced pT3 and pT4 adenocarcinoma with intratumoral or peritumoral invasion to the vasculature, perineural invasion, or metastasis in lymph nodes (Figure 1c–e). As a result, our patient data suggested that ERK1/2 activation contributes to the migration or invasion of colon cancer cells.

### 3.2. ERK1 and ERK2 Expression In Vitro Models

**Figure 2:**
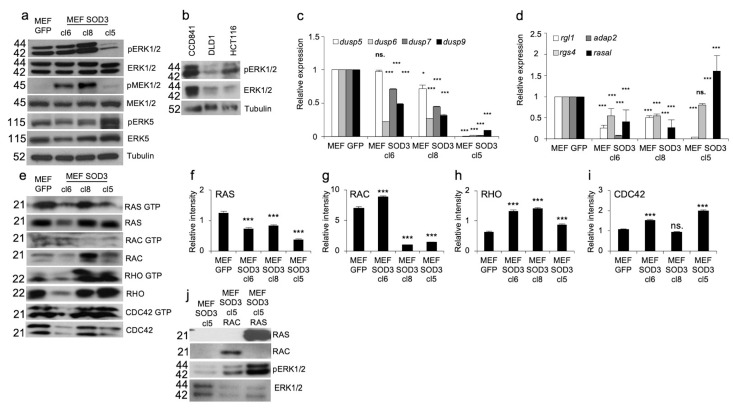
Characterization of ERK1/2 expression in vitro models. (**a**) Western blot analysis of MEF GFP, MEF SOD3 cl6, MEF SOD3 cl8, and MEF SOD3 cl5. Our analysis demonstrated moderately increased ERK1/2 phosphorylation in cl8 and markedly decreased phosphorylation in cl5. MEK1/2 staining supported the ERK1/2 activation results. The phosphorylation of ERK5 was moderately increased in MEF SOD3 cl5 cells. (**b**) Western blot analysis for ERK1/2 phosphorylation in CCD841, DLD1, and HCT116 cells. Both the phosphorylation and total ERK1/2 levels are lower in DLD1 and HCT116 adenocarcinoma cells as compared to CCD841 normal colon epithelial cells. (**c**) The mRNA expression analysis for *dusp5*, *6*, *7*, and *9* showed significantly decreased expression in all MEF SOD3 clones. (**d**) The mRNA expression analysis for small GTPase regulatory gene expression showed decreased *rgl1*, *rgs4*, *adap2*, and *rasal* mRNA levels in MEF SOD3 cl6 and cl8. The MEF SOD3 cl5 *adap2* expression was at the same level as in MEF GFP controls, while the *rasal* expression was significantly increased. (**e**) Pull down assay for RAS, RAC, CDC42, and RHO in MEF GFP, MEF SOD3 cl6, MEF SOD3 cl8, and MEF SOD3 cl5. (**f**–**i**) Intensity measurement of small GTPase pulldown assay. (**j**) RAS, RAC, and ERK1/2 Western blot in MEF SOD3 cl5 cell overexpressing *ras* or *rac*. *p*-values are * *p* < 0.05, and *** *p* < 0.001. *p*-value was determined using *GFP*-expressing cells as a comparison control. ns. refers to non-significant.

Mitogen signal transduction can be mediated by ROS, such as SOD3-produced H_2_O_2_, which modifies the activation of phosphotyrosine phosphatases (PTPs) [31], small GTPases [16], and their downstream kinases. To study the SOD3-related signaling involved in ERK1/2 phosphorylation we used a mouse embryonic fibroblast (MEF) model composed of MEF clones cl6, cl8, and cl5 with a GFP control [25] demonstrating downregulation of ERK1/2 phosphorylation in cl5 with corresponding upregulation of ERK5 activation in MEF SOD3. The Western blot analysis further suggested moderately upregulated MEK1/2 and ERK1/2 activation in the cl8 (Figure 2a), confirming previous results demonstrating a regulatory role for the enzyme in mitogen signaling [16]. Analysis of the expression and activation of ERK1/2 in CCD841 normal colon cells, DLD1 colon adenocarcinoma cells, and aggressive HCT116 colon carcinoma cells suggested downregulation of both the phosphorylation and the expression of total ERK1/2 proteins in DLD1 and HCT116 cells (Figure 2b).

To dissect the factors regulating ERK1/2 phosphorylation in SOD3 overexpressing cells, we first focused on the expression of *dual specific phosphatases* (*dusp*s) *5, 6, 7,* and *9*, which regulate activation of the mitogen-activated protein kinase (MAPK) family, especially ERK1/2 kinases.

As shown in Figure 2c, the mRNA expression of the studied *dusp*s was not increased in MEF SOD3 clones, thereby suggesting that other factors were causing the reduced ERK1/2 phosphorylation in MEF SOD3 cl5 (Figure 2c). Analysis of the expression of small GTPase and heterotrimeric G protein regulatory genes *RGL1* (*Ral guanine nucleotide dissociation stimulator-like 1*), *adap2* (*ArfGAP with dual PH domains 2*), *rgs4* (*regulator of G protein signaling 4*), and *rasal* (*RAS GAP activating protein*) demonstrated significantly (*p* > 0.001) upregulated *rasal* mRNA synthesis in MEF SOD3 cl5 (Figure 2d). The data, therefore, suggested the contribution of the small GTPase RAS regulatory protein GAP in the downregulation of ERK1/2 phosphorylation.

Next, to analyze the activation status of small GTPases, we performed a pull-down assay for RAS, RAC, RHO, and CDC42, demonstrating a significant (*p* > 0.001) downregulation of RAS GTP loading in all MEF SOD3 clones (Figure 2e–i). RAC activation was upregulated in MEF SOD3 clone 6 and significantly downregulated in clones 8 and 5. RHO was significantly (*p* < 0.001) upregulated in all clones and CDC42 in clones 6 and 5, respectively (Figure 2e–i). To further characterize the contribution of SOD3 on ERK1/2 activation, we studied the effect of small GTPase RAC and RAS transient overexpression on the activation of ERK1/2 in the MEF SOD3 cl5 (Figure 2j). RAS had a pronounced role in the phosphorylation of ERK1/2 kinases, although RAC also stimulated the activation, suggesting parallel RAS and RAC signaling in ERK1/2 activation. 

### 3.3. ERK1 and ERK2 Have Different Functions in Cell Migration and Proliferation In Vitro

**Figure 3:**
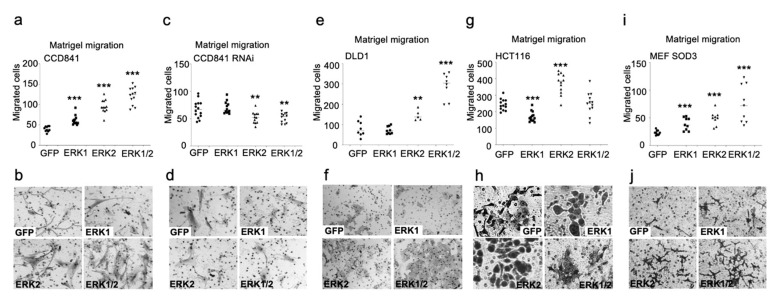
Chemotactic invasion through Matrigel. Characterization of the effect of *ERK1/2* overexpression on migration in vitro in CCD841 (**a**,**b**), CCD841 RNAi (**c**,**d**), DLD1 (**e**,**f**), HCT116 (**g**,**h**), and MEF SOD3 cells (**i**,**j**). According to the results, *ERK2* overexpression resulted in a higher invasion rate as compared to *ERK1* overexpression. RNAi significantly reduced the invasion potential of *ERK2* and *ERK1/2* cells below GFP control cells. *p*-values are ** *p* < 0.01, and *** *p* < 0.001. The *p*-value was determined using *GFP*-expressing cells as a comparison control. Magnification of the cells is 20×.

Next, we studied the individual effects of ERK1 and ERK2 kinases in the colon in vitro models. Chemotactic invasion assay suggested different capacities for ERK1 and ERK2 to promote cellular invasion towards chemo-attractants through the extracellular matrix (Figure 3a–j). In normal colon CCD841 cells, both *ERK1* and *ERK2* supported cell migration (*p* < 0.001), which was further enhanced by the combined expression of *ERK1/2* (*p* < 0.001) (Figure 3a,b). RNA interference abolished the stimulatory effect of *ERK1* and significantly (*p* < 0.01) decreased invasion in ERK2 and ERK1/2 cells as compared to GFP control cells (Figure 3c,d). In DLD1 cells *ERK1* demonstrated a complete deficiency to promote invasion, whereas *ERK2* significantly (*p* < 0.01) stimulated invasion, which was further enhanced by simultaneous *ERK1* and *ERK2* expression similar to CCD841 cells (Figure 3e,f). In HCT116 cells, *ERK2* demonstrated increased migration potential (*p* < 0.001), whereas *ERK1* and *ERK1/2* invasion was at the level of control cells (Figure 3g,h). To study the effect of *ERK1* and *ERK2* in MEFs, we used MEF SOD3 cl5 cells, which we termed MEF SOD3 GFP, MEF SOD3 ERK1, MEF SOD3 ERK2, or MEF SOD3 EKR1/2. Our analysis of MEF clones further corroborated the results suggesting a more prominent role for *ERK2* in the stimulation of invasion, which was further enhanced by simultaneous expressions of *ERK1* and *ERK2* (Figure 3i,j).

**Figure 4:**
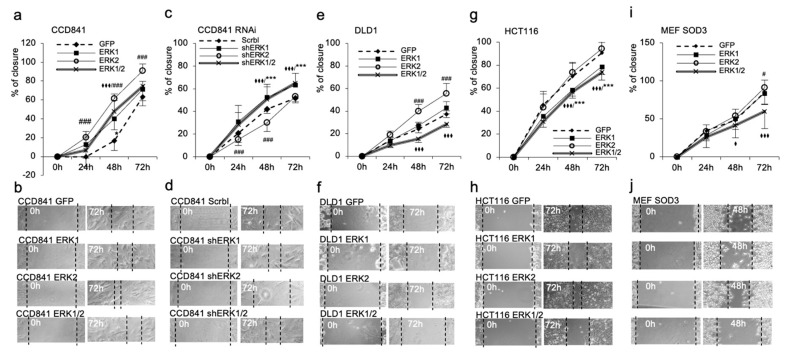
Collective cell migration in wound healing assay. *ERK2* overexpression promoted significantly higher migration in CCD841 (**a**,**b**), DLD1 (**e**,**f**), and MEF SOD3 (**i**,**j**) cells as compared to *GFP*-expressing controls. *ERK1* overexpression had an obsolete effect on cell movement, whereas simultaneous *ERK1* and *ERK2* overexpression reduced collective cell migration in DLD1 (**e**,**f**), HCT116 (**g**,**h**), and MEF SOD3 cells (**i**,**j**). RNAi for *ERK1* and *ERK2* in CCD841 cells (**c**,**d**) significantly reduced the collective migration of shERK2 cells below the Scrbl control cells, whereas it significantly increased the migration of shERK1/2 cells. *p*-values are ^#/♦^ *p* < 0.05, and ***^/###/♦♦♦^ *p* < 0.001. The *p*-value was determined using *GFP/Scrbl* expressing cells as a comparison control. (*/*** refers to ERK1, #/### refers to ERK2, ♦/♦♦♦ refers to ERK1/2).

The collective cell migration assay, which regulates cellular movement as a structural and functional unit [32], corroborated the more prominent function of ERK2. *ERK2* overexpression demonstrated faster scratch recovery in all time points as analyzed in CCD841, DLD1, HCT116, and MEF SOD3 when compared to *ERK1* expressing cells, which did not affect cell migration (Figure 4a,b,e–j). RNA interference in CCD841 cells decreased the migration capacity of ERK2 cells below the control cells, although it significantly (*p* < 0.001) increased the collective migration of *ERK1* and *ERK1/2* expressing cells (Figure 4c,d).

It is noteworthy that the combined expression of *ERK1* and *ERK2* in DLD1, HCT116, and MEF SOD3 cells significantly (*p* < 0.001) reduced cellular movement as compared to *GFP*-transduced controls (Figure 4i,j).

**Figure 5:**
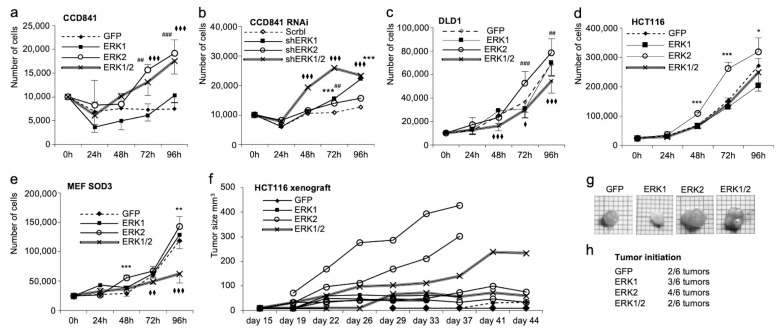
Characterization of the effect of *ERK1/2* overexpression on cell proliferation in vitro and in vivo in CCD841, CCD841 RNAi, DLD1, HCT116, and MEF SOD3 cells. (**a**–**e**) *ERK2* overexpression significantly increased cell proliferation in all cell models studied, whereas *ERK1* overexpression had an obsolete effect. Simultaneous *ERK1/2* expression promoted cell proliferation in CCD841 cells, whereas it inhibited growth in DLD1 and MEF SOD3 cells. Similar to collective migration, RNAi significantly reduced cell proliferation of shERK2 cells and increased shERK1/2 cells. (**f**) In vivo tumorigenesis of HCT116 cells overexpressing *GFP*, *ERK1*, *ERK2*, or *ERK1/2* in nude mice. *ERK2* overexpressing cells demonstrated the highest tumor formation capacity corroborating in vitro data. (**g**,**h**) Tumor size and tumor initiation capacity further strengthened the obtained results suggesting moderately reduced tumor sizes in *ERK1* overexpressing cells and highest tumor sizes in *ERK2* expressing cells. The tumor formation capacity data showed two tumors out of six transplantations for *GFP* control cells, three tumors out of six transplantations for *ERK1* cells, four tumors out of six transplantations for *ERK2* cells, and two tumors out of six transplantations for *ERK1/2* cells. *p*-values are */^♦^ *p* < 0.05, **/^##^/^♦♦^ *p* < 0.01, and ***/^###^/^♦♦♦^ *p* < 0.001. The *p*-value was determined using *GFP* expressing cells as a comparison control. (*/**/*** refers to ERK1, ^##^/^###^ refers to ERK2, ^♦^/^♦♦^/^♦♦♦^ refers to ERK1/2).

Our analysis of cell proliferation further strengthened the tumor-promoting role of ERK2 in cancer cells. *ERK2* overexpression resulted in a higher proliferation rate when compared to the overexpression of *ERK1* or *GFP* control gene in all cell lines studied (Figure 5a,c–e). The combined overexpression of *ERK1* and *ERK2* increased growth in CCD841 normal colon cells (Figure 5a) but decreased the growth in DLD1 and MEF SOD3 cultures when compared to the *GFP* controls (Figure 5c,e), which is similar to collective cell migration (Figure 4e,f,i,j). RNAi in CCD841 cells eliminated the growth stimulatory role of *ERK2* and increased cell proliferation in ERK1 and ERK1/2 cells as compared to control cells (Figure 5b).

Previous reports have suggested that ERK1/2 could influence the differentiation of the cells, such as PKC-Ca^2+^-stimulated differentiation of epidermal keratinocytes and stem cell factor-erythropoietin-mediated maturation and expansion of erythroid progenitor cells [9,33,34]. To dissect the function of ERK1 in colon tumorigenesis, we analyzed *OCT4*, *VIMENTIN*, *CHI3*, and *CDX2* differentiation marker mRNA expression. According to our analysis, *ERK1* overexpression does not promote differentiation of DLD1 cells (Supplemental Figure S2).

To confirm the data showing the variable growth potential of *ERK1* and *ERK2* expressing cells, we injected 1 × 10^6^ HCT116 cells subcutaneously in BALB c/A nude mice. HCT116 cells expressing *ERK2* cells resulted in larger tumors as compared to *ERK1* or *ERK1/2* cells, thereby validating the in vitro data (Figure 5f,g). Besides their increased tumorigenic potential, *ERK2* cells had a higher incidence of tumor initiation: 66% (4 out of 6 injections) of HCT116 *ERK2* transplantations resulted in detectable tumors, whereas 50% (3 out of 6 injections) of HCT116 *ERK1* cells, 33% (2 out of 6 injections) of HCT116 ERK1/2 cells transplantations, and 33% (2 out of 6 injections) of HCT116 GFP cells yielded tumors (Figure 5h).

## 4. Discussion

The ability of RAS-ERK1/2 signaling to coordinate diverse cellular processes is essential for different biological functions, including ontogenesis [35]. In cancer, ROS, predominantly H_2_O_2_, along with O_2_^−^, coordinate cell signaling, tumor initiation, oncogene-induced senescence, and benign to malignant transformation [13,14,36,37,38]. ROS and redox enzymes, such as SOD3, are involved in a variety of cellular phenomena ranging from DNA damage and point mutations to tissue-level metabolic disorders [29,39,40,41]. SOD3, by balancing the local concentrations of O_2_^−^ and H_2_O_2_, forms a positive feedback loop to regulate the RAS-MEK1/2-ERK1/2 signaling pathway [15] through the coordination of guanine nucleotide exchange factor (GEF), GTPase activating protein (GAP), and guanosine nucleotide dissociation inhibitor (GDI) expression [16]. The current data demonstrating SOD3-derived downregulation of ERK1/2 phosphorylation (Figure 2) suggest the involvement of H_2_O_2_ in the coordinated interaction of RAS, ERK1/2, and ERK5 in colon tumorigenesis.

ERK1 and ERK2 kinases are generally co-phosphorylated by the same extracellular stimuli, although the kinases are suggested to have different biological functions ranging from severe abnormality of the placenta, with subsequent embryonic lethality [42], to a distinct expression pattern in the adult murine brain [43]. After the activation, ERK1 and ERK2 are generally translocated to the nucleus, although they can also be detected at the cytoplasmic compartment, e.g., at Golgi and the cell membrane [44,45]. The inactivation of ERK1 and ERK2 isoform phosphorylation in the early phase of colon tumorigenesis, refs. [19,20,21] with subsequent increased phosphorylation of ERK5 [22], is an intriguing phenomenon proposing an inhibitory function for ERK1/2 in the initial phases of adenocarcinoma development. Another ambiguous phenomenon is the activation of ERK1/2 kinases in metastatic patients (Figure 1).

In the current work, we demonstrated a more pronounced role for *ERK2* than for *ERK1* in promoting chemotactic invasion, collective migration, and proliferation (Figure 3, Figure 4 and Figure 5). Notably, *ERK1* alone had only a minor, if any, stimulatory role on cellular functions or differentiation marker expression (Supplemental Figure S2), whereas the simultaneous overexpression of *ERK1* and *ERK2* reduced collective migration and cell proliferation. The reduced cellular growth caused by *ERK1/2* transduction could be a growth-limiting factor in the early phase of tumor expansion and therefore a reason for the downregulation of ERK1/2 phosphorylation in colon adenomas. The loss of ERK1/2 phosphorylation at the early benign phase of colon tumorigenesis [19,20,21] with subsequent ERK5 activation [22] could provide newly transformed cancer cells with a growth advantage.

ERK1/2 have at least 49 direct nuclear and cytoplasmic downstream substrates, mostly transcriptional factors Ets family members, including Ets-1 and Elk1, Smad proteins, c-Fos, c-Myc, and ATF2, and a markedly higher number of indirect targets, which, as a network, coordinate cellular functions [46,47]. Therefore, the decreased cell proliferation of simultaneous *ERK1/2* expression as demonstrated in our data could result from the inhibition of nuclear translocation of the kinases. Entry into the cell cycle, G1 to S phase progression occurs only after nuclear translocation of phosphorylated ERK1/2 that stimulates ELK1 transcription and reinitiation of DNA replication [48,49], which then activates the DNA replication machinery.

Another plausible explanation for reduced growth and collective cell migration caused by simultaneous *ERK1* and *ERK2* expression could be the inhibition of dimerization of ERKs, which was reported to halt cell proliferation. Dimerization enhances ligand binding in the nucleus with subsequent entry to cell cycling. Correspondingly, inhibition of dimerization inhibits the loss of cellular differentiation, growth, and tumorigenesis [45,50].

Our present data showing increased chemotactic invasion in cells transduced with both *ERK1* and *ERK2* kinases (Figure 3a–j) may suggest that the kinases are needed for the initiation and progression of metastasis in colon carcinogenesis. This is supported by a previous article suggesting that *KRAS* and *BRAF* mutations with consequent activation of MEK1/2-ERK1/2 signaling increase the risk of lung metastasis in colorectal cancer patients [51]. A recent work consolidates the current results by proposing an increased risk of colon cancer liver metastasis caused by the ERK1/2-stimulated upregulation of *ANGPT2* and *CXCR4* [52], thereby proving a mechanism for how ERK1/2 signaling may promote colon cancer cell invasion.

According to our results, *ERK1* expressed alone had a minor or no role in colon cancer behavior but, together with *ERK2* expression, the kinase pair had an impact on in vitro invasion, migration, proliferation, and in vivo tumorigenesis. Correspondingly, RNA interference of both *ERK1* and *ERK2* in CCD481 cells abolished the ability of cells to invade through the extracellular matrix and significantly increased collective migration and cell proliferation as compared to CCD841 cells overexpressing *ERK1* and *ERK2*. Therefore, ERK1 could be a regulatory kinase coordinating the activity of ERK2 either by enhancing or inhibiting the ERK2 response in the cells.

In conclusion, herein, we demonstrated that in colon adenocarcinoma in vitro and in vivo models, ERK2 stimulates migration and proliferation, while ERK1 alone has minor or no effect on cellular functions. Simultaneous *ERK1/2* expression in cancer cells markedly reduced colon cancer cell proliferation and tumor formation, thereby suggesting that ERK1 regulates ERK2. The growth disadvantage of ERK1/2-expressing cells could explain the inhibition of the kinase phosphorylation in the early stages of colon tumorigenesis. Consequently, more developed and specific ERK2-targeted small molecule inhibitors mimicking the function of ERK1 may ameliorate primary tumor expansion. Our observed correlation between re-phosphorylation of the kinases and intratumoral migration or metastasis may imply that the kinases could theoretically be a potential short-term drug target to inhibit colon cancer metastasis.

## Data Availability

Data is contained within the article and Supplementary Materials.

## Supplementary Materials

The following supporting information can be downloaded at: https://www.mdpi.com/article/10.3390/antiox13010119/s1, Figure S1: STRs of cell CCD841, DLD1, and HCT116 showing marked differences between cell lines; Figure S2: OCT4, VIMENTIN, CHI3, and CDX2 differentiation marker expression in DLD1 cells expressing GFP, ERK1, ERK2, or ERK1/2; Table S1: PCR primers.
