# Supplementary material for: ERK2 Is a Promoter of Cancer Cell Growth and Migration in Colon Adenocarcinoma
Source: Antioxidants (Basel). 2024 Jan 17;13(1):119. doi: 10.3390/antiox13010119 (PMC10812609; doi:10.3390/antiox13010119)
Supplement: Supplementary file 1 [file antioxidants-13-00119-s001.zip › antioxidants-2773283-supplementary.pdf]

Supplemental Table S1. PCR primers.

Mouse DUSP5 forward	acagaccagcctatgaccag
Mouse DUSP5 reverse	cacggggatccacttgtagt
Mouse DUSP6 forward	gttcctcttgagcagcatcg
Mouse DUSP6 reverse	agcatgtcctgtccttccaa
Mouse DUSP7 forward	gccaaggactctaccaacct
Mouse DUSP7 reverse	cggggaagaactgagagagg
Mouse DUPS9 forward	ccactacaagcagatcccca
Mouse DUSP9 reverse	ccaagtcgtaggcatcgttg
Mouse RGL1 forward	agctgctgctggacaggtat
Mouse RGL1 reverse	ctgaaggcaagggaagtgag
Mouse RGS4 forward	gctaaggggtgagcactctg
Mouse RGS4 reverse	tctgccctcacctaagcagt
Mouse ADAP2 forward	gtgctccttccagcaaactc
Mouse ADAP2 reverse	ctgaactgaacccccagtgt
Mouse RASAL forward	tgtagggtcacctcctggtc
Mouse RASAL reverse	ttggtcatccactttcacca
Mouse 18S rRNA forward	agaaacggctaccacatcca
Mouse 18S rRNA reverse	ccctccaatggatcctcgtt
Mouse ERK1 forward	tgggccaagctctttcctaa
Mouse ERK1 reverse	agctccatgtcgaaggtgaa
Mouse ERK2 forward	ccaacaggcctatcttccca
Mouse ERK2 reverse	acctgttccatggcacctta
Human ERK1 forward	tggcaagcactacctggatcag
Human ERK1 reverse	gcagagactgtaggtagtttcggg
Human ERK2 forward	gtgacctcaagccttccaac
Human ERK2 reverse	agaatgcagcctacagacca
Human Oct4 forward	ggtccgagtgtggttctgta
Human Oct4 reverse	cgaggagtacagtgcagtga
Human VIMENTIN forward	gagtccactgagtaccggag
Human VIMENTIN reverse	acgagccatttcctccttca
Human Chi3 forward	ttccctctcaccaatgccat
Human Chi3 reverse	agggctgagctcaaatctgt
Human CDX2 forward	ggacgtgagcatgtacccta
Human CDX2 reverse	gtagccattccagtcctccc
Human beta actin forward	tgcgttgacattaaggagaag
Human beta actin reverse	gctcgtagctcttctcca
Human 18S RNA forward	gttggttttcggaactgagg
Human 18S RNA reverse	gcatcgtttatggtcggaac

CCD841	DLD1	HCT116
Amelogenin: X	Amelogeni: X,Y	Amelogenin: X,Y
CSF1PO: 10,11	CSF1PO: 1,12	CSF1PO: 7,10
D13S317: 11,13	D13S317: 8,11	D13S317: 10,12
D16S539: 10,11	D16S539: 12,13	D16S539: 11,13
D5S818: 12,13	D5S818: 13	D5S818: 10,11
D7S820: 11	D7S820: 10,12	D7S820: 11,12
THO1: 7,8	TH01: 7,9.3	TH01: 8,9
TPOX: 9,10	TPOX: 8,11	TPOX: 8,9
vWA: 14,18	vWA: 18,19	vWA: 17,22

Supplemental Figure S1. STRs of cell CCD841, DLD1, and HCT116 showing marked differences between cell lines.

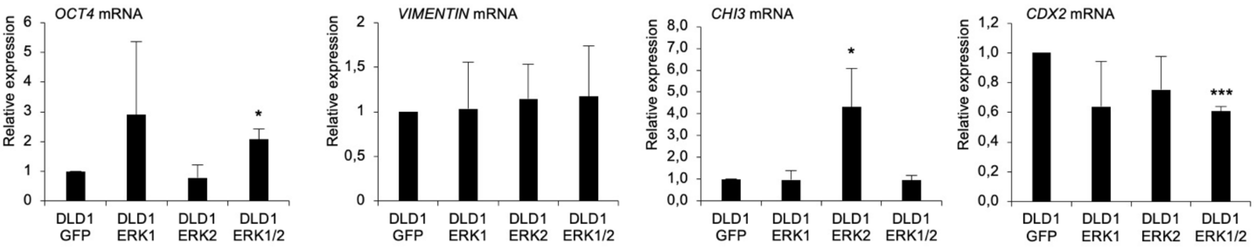

Supplemental Figure S2. *OCT4*, *VIMENTIN*, *CHI3*, and *CDX2* differentiation marker expression in DLD1 cells expressing *GFP*, *ERK1*, *ERK2*, or *ERK1/2*. The data did not show any effect for ERK1 in the differentiation of the cells. The p-values are *p<0.05, **p<0.01, ***p<0.001, and ****p<0.0001. The p-value was determined using *GFP* expressing cells as a comparison control.
